# Impact of a Virtual Reality Video ("A Walk-Through Dementia") on YouTube Users: Topic Modeling Analysis

**DOI:** 10.2196/67755

**Published:** 2025-04-16

**Authors:** Xiaoli Li, Xiaoyu Liu, Cheng Yin, Sandra Collins, Eman Alanazi

**Affiliations:** 1 School of Health Sciences, Southern Illinois University Carbondale, 1365 Douglas Drive, ASA Building - Mail Code 6615, Carbondale, IL, 62901, United States, 1 9407583557; 2 College for Public Health & Social Justice, Saint Louis University, Saint Louis, MO, United States; 3 Department of Rehabilitation and Health Services, University of North Texas, Denton, TX, United States; 4 Health Informatics Department, College of Health Sciences, Saudi Electronic University, Riyadh, Saudi Arabia

**Keywords:** Alzheimer disease, Alzheimer disease and related dementias, ADRD, dementia, public awareness, text mining, older adult, health care student, training, health care professional, simulation, digital world, virtual environment, virtual tour, walk-through dementia, virtual reality, VR, VR video, VR application, topic modeling, YouTube, Bidirectional Encoder Representations from Transformers, BERT, social media comments, experiential learning tool

## Abstract

**Background:**

Emerging research has highlighted the potential of virtual reality (VR) as a tool for training health care students and professionals in care skills for individuals with Alzheimer disease and related dementias (ADRD). However, there is limited research on the use of VR to engage the general public in raising awareness about ADRD.

**Objective:**

This research aimed to examine the impact of the VR video “A Walk-Through Dementia” on YouTube users by analyzing their posts.

**Methods:**

We collected 12,754 comments from the VR video series “A Walk-Through Dementia,” which simulates the everyday challenges faced by individuals with ADRD, providing viewers with an immersive experience of the condition. Topic modeling was conducted to gauge viewer opinions and reactions to the videos. A pretrained Bidirectional Encoder Representations from Transformers (BERT) model was used to transform the YouTube comments into high-dimensional vector embeddings, allowing for systematic identification and detailed analysis of the principal topics and their thematic structures within the dataset.

**Results:**

We identified the top 300 most frequent words in the dataset and categorized them into nouns, verbs, and adjectives or adverbs using a part-of-speech tagging model, fine-tuned for accurate tagging tasks. The topic modeling process identified eight8 initial topics based on the most frequent words. After manually reviewing the 8 topics and the content of the comments, we synthesized them into 5 themes. The predominant theme, represented in 2917 comments, centered on users’ personal experiences with the impact of ADRD on patients and caregivers. The remaining themes were categorized into 4 main areas: positive reactions to the VR videos, challenges faced by individuals with ADRD, the role of caregivers, and learning from the VR videos.

**Conclusions:**

Using topic modeling, this study demonstrated that VR applications serve as engaging and experiential learning tools, offering the public a deeper understanding of life with ADRD. Future research should explore additional VR applications on social media, as they hold the potential to reach wider audiences and effectively disseminate knowledge about ADRD.

## Introduction

With a rapidly aging population, the number of older adults living with chronic conditions is on the rise. Among these conditions, Alzheimer disease and related dementias (ADRD) have become leading causes of disability and dependency worldwide, characterized by memory loss and other cognitive impairments [1]. In the later stages, individuals with ADRD may lose the ability to interact with their surroundings, making care management increasingly complex both within health care settings and at home [2]. This condition not only affects the quality of life for those diagnosed but also places a significant burden on health care systems and families. While most older adults prefer to stay in their homes as long as possible, many eventually require higher levels of care, often transitioning to nursing homes or memory care facilities [3]. Caregivers—whether family members or professionals—must have a thorough understanding of ADRD to provide the best care possible. In addition, various policy initiatives, such as those promoting the creation of “dementia-friendly communities” [4], underscore the importance of raising public awareness about dementia. These communities aim to foster environments where people understand ADRD and where individuals with dementia feel included, involved, and empowered to maintain control over their daily lives [5]. Such initiatives emphasize the need for widespread dementia awareness and education within communities to improve the support and care provided to those affected by ADRD.

However, there is a lack of public awareness and understanding of what ADRD is and its impact on individuals’ abilities, well-being, and capacity to live meaningful lives [6]. As a result, misunderstanding and discrimination among people living with ADRD are common. For instance, there are false beliefs that people with ADRD cannot experience quality of life, are entirely dependent on others, and have lost all autonomy and dignity [6]. Such stigma and misunderstandings can have negative effects on the psychosocial well-being, self-esteem, and overall quality of life for both those living with ADRD and their caregivers. In addition, stigma can contribute to social exclusion, isolation, and even self-stigmatization, further exacerbating the challenges faced by individuals with ADRD and their families [7].

Disseminating reliable information about ADRD is especially crucial because addressing modifiable factors can help reduce misunderstandings, provide support to caregivers, and raise awareness about the condition [8,9]. Social media platforms, particularly YouTube, have become powerful tools for disseminating health-related information to audiences that are not easily accessible [10]. With 73% of middle-aged adults and 45% of older adults in the United States using social media such as Facebook (Meta) and YouTube, these platforms are vital tools for raising awareness, educating communities, and deepening public understanding of ADRD [11]. They not only facilitate the sharing of personal stories but also provide educational content, making them well-positioned to increase knowledge and combat the stigma associated with ADRD [12].

Previous studies have explored various topics involving YouTube and ADRD, such as assessing the quality of ADRD caregiving information on the platform [13], describing social media activities for aging and ADRD education [14], developing internet-based ADRD training modules [15], examining YouTube’s effectiveness in dementia education to a target community [16], and exploring the role of personal stories in raising awareness about dementia [17]. In addition, research has examined the role of personal stories in raising awareness about dementia and how social media mobilizes knowledge about pain management in dementia [18]. However, these studies focused on traditional videos as educational tools, and no research has examined the use of virtual reality (VR) videos for public ADRD education.

VR videos provide immersive, 360-degree environments that allow users to engage with simulated worlds using devices like enclosed headsets or body suits [19,20]. In the field of ADRD education, unlike traditional media, VR offers a unique opportunity to simulate the experiences of individuals living with dementia, providing a more visceral understanding of their challenges. While VR has primarily been used to educate health care students and the caregiving workforce about ADRD [21-23], its potential for raising public awareness has not been fully explored. This gap is particularly important, as VR can deliver an engaging and experiential form of learning that may influence attitudes towards ADRD.

This study aims to explore the impact of a free YouTube VR video series titled “A Walk-through Dementia” (AWTD), which simulates the experiences of individuals living with dementia [20]. AWTD consists of 3 videos, each lasting 3 to 5 minutes, and was developed by Alzheimer’s Research United Kingdom to enhance understanding of the challenges faced by people with dementia. This research seeks to understand the general public’s experience with VR videos on ADRD. By analyzing YouTube comments, the study will explore whether these VR videos can enhance public awareness of ADRD and serve as an effective tool for addressing misunderstandings and stigma surrounding ADRD.

## Methods

### Overview

**Figure 1.**
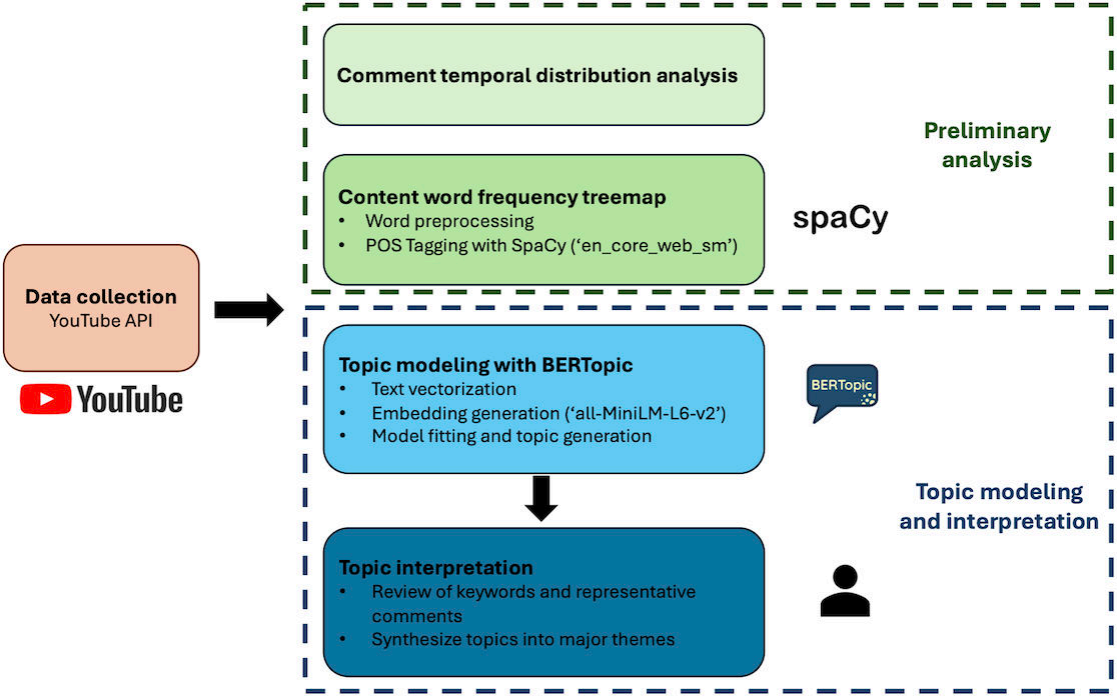
The workflow of the methods. It includes data collection via the YouTube API, preliminary analysis, and topic modeling using BERTopic. The final stage involves interpreting topics by reviewing keywords and synthesizing them into major themes. API: application programming interface; BERT: Bidirectional Encoder Representations from Transformers.

We followed a 3-step exploratory research approach to conduct the study. First, we collected comprehensive data by gathering the most viewed VR video series on YouTube. Second, we performed topic modeling in the comments section to identify the main themes discussed. Third, we used Thematic analysis to conduct these themes into concise, easily digestible summaries. The workflow of the methods is outlined in Figure 1 below.

### Datasets

The analyzed video comments are from a series of 360° videos titled “A Walk-Through Dementia,” the most viewed videos under the search terms “dementia” and “Alzheimer disease.” Developed by Alzheimer’s Research United Kingdom, this series aims to raise awareness about the impact of dementia on individuals’ lives by engaging the public, health care professionals, and caregivers. The series comprises 3 main scenarios depicted in separate videos (Textbox 1) .

Textbox 1. **Three main scenarios in “A Walk-Through Dementia.”**

- At the supermarket: the video demonstrates how dementia affects tasks such as reading shopping lists, dealing with short-term memory loss, changes in food preferences, and managing social anxiety at checkout counters.
- Walking home: the video highlights the difficulties in navigating busy streets, recognizing familiar places and people, and managing visual and spatial misperceptions.
- At home: the video illustrates challenges within a familiar environment, such as difficulty remembering instructions, issues with hand-eye coordination, and managing multi-step tasks like making a cup of tea.

Compared to other VR videos retrieved using the same search terms, the AWTD series has garnered the highest view counts (walking home [WH]: 3.2 million views; at the supermarket [AS]: 2.7 million views; at home [AH]: 0.12 million views) relative to the most viewed videos by other creators (eg, “Coping with Alzheimer’s, Together and Apart” with 67 thousand views). In addition, this series has attracted the most comments. To maintain content consistency and consider the low number of comments in other VR videos (eg, “Coping with Alzheimer’s, Together and Apart” with 66 comments), we decided to focus solely on the comment sections of the 3 videos in this series.

### Preliminary Analysis

To collect the comments for this study, we used the official YouTube application programming interface, retrieving both primary comments and their corresponding replies. This resulted in a total of 12,754 comments. After removing duplicate and irrelevant entries, the final dataset included 11,543 unique comments.

Before conducting topic modeling, we performed a series of preliminary analyses to gain an initial understanding of the content across the 3 videos. These analyses involved examining the temporal distribution of comment postings and calculating word frequencies. The purpose of this step was to identify key content words essential for interpreting the overarching themes in the comments.

To ensure focus on the most meaningful words, we restricted our analysis to content words, specifically nouns, main verbs, adjectives, and most adverbs. We identified the top 300 most frequent words in the dataset and categorized them into nouns, verbs, and adjectives or adverbs using a part-of-speech tagging model, fine-tuned for accurate tagging tasks. In addition, we manually corrected any miscategorized words during the automated tagging process. The frequencies of these words were calculated and used to create a treemap visualization, highlighting the distribution and prominence of these content words across the comments.

### Topic Modeling With BERTopic

Topic modeling is widely used in health informatics and related disciplines for text mining large datasets, such as comments or tweets, to extract main topics from extensive texts. For our study, we used BERTopic (Bidirectional Encoder Representations from Transformers), a state-of-the-art topic modeling technique that leverages transformer-based deep learning models to identify topics in large text collections. Its powerful capabilities have been demonstrated in various studies [24-26]. It consistently outperforms traditional topic modeling methods, such as latent Dirichlet allocation (LDA) and nonnegative matrix factorization (NMF), with a particularly pronounced advantage in analyzing short and unstructured texts, where embedding-based models show greater potential for generating meaningful insights [27]. To validate this choice, we conducted empirical comparisons between BERTopic, LDA, and NMF using topic diversity scores, which measure the uniqueness of generated topics. It is calculated as the ratio of the number of unique words to the product of the number of top words per topic and the total number of topics [28]. BERTopic achieved a significantly higher score (0.8750) compared to LDA (0.2375) and NMF (0.25), indicating its superior ability to produce distinct and less redundant topics. These results align with previous research, which highlights BERTopic’s effectiveness in capturing contextual meaning and semantic nuances often missed by traditional models [27,29]. All analyses and visualizations were conducted using Python in Colab, using the BERTopic package (version 0.16.0).

We began by preprocessing the text data and removing special characters, symbols, and punctuation. Then, we used the vectorization technique CountVectorizer to convert the preprocessed text into numerical representations. These numerical representations capture the frequency or importance of words in each document and can be directly input into topic modeling algorithms.

Subsequently, we conducted topic modeling with BERTopic. Based on a preliminary review of the comments, we estimated the number of topics to range from 5 to 10. After training models with various predefined topic numbers, we selected 8 as the final number because it provided a balance between maintaining model coherence and capturing meaningful thematic distinctions within the comments and retained only topics that included more than 50 comments. As part of the topic modeling process, we analyzed both unigrams and bigrams to generate topics. While unigrams offer a broad sense of the topics by identifying the most frequent individual terms, bigrams provide a more fine-grained perspective by highlighting word pairs that are often used together [30]. Analyzing both unigrams and bigrams helped create a more comprehensive understanding of the topics discussed.

Topic modeling is a common approach to analyzing social media content, such as Facebook (Meta) posts, tweets, and Reddit communities, related to public health. It identifies key themes, including awareness campaigns, personal caregiving experiences, and scientific advancements [31]. This approach provides a framework for understanding how structured health campaigns influence public discourse on social media [32]. Therefore, topic modeling is suitable for this study to examine comments on VR videos on YouTube, contributing to a more comprehensive view of ADRD in social media contexts.

### Ethical Considerations

This study consisted of observations of public behaviors using secondary research data. The study data are anonymous and deidentified. Therefore, ethical approval was not required.

## Results

### Overview of the Video Comments

We extracted all comments from the 3 VR videos in the AWTD series. Both WH and AH were posted on June 2, 2016, while AS was posted the following year on March 21, 2017. As of September 2024, when the data was collected, AS had the highest number of comments, with 6572 (including replies), WH had 5954 comments, and AH had 122 comments.

The treemap visualization illustrates the most frequently used words in the comments across the 3 videos. Each rectangle represents a word, with its size and color intensity corresponding to the word’s frequency in the comments for each video. The word “dementia” is the most prominent in the noun category, indicating that it is a central topic in the discussions. Other significant nouns include “family,” “home,” “memory,” “people,” “video,” “mom,” and “disease.” These nouns suggest that the comments often focus on personal experiences, caregiving, videos as a tool, and the impact of dementia on individuals and families.

Among the verbs, words including “know,” “remember,” “think,” and “lost” are frequent. This shows that the comments are action-oriented, reflecting emotions, actions taken, or care experiences of the commenters. The presence of verbs like “forgot” and “care” suggests a focus on ADRD symptom and caregiving actions.

**Figure 2.**
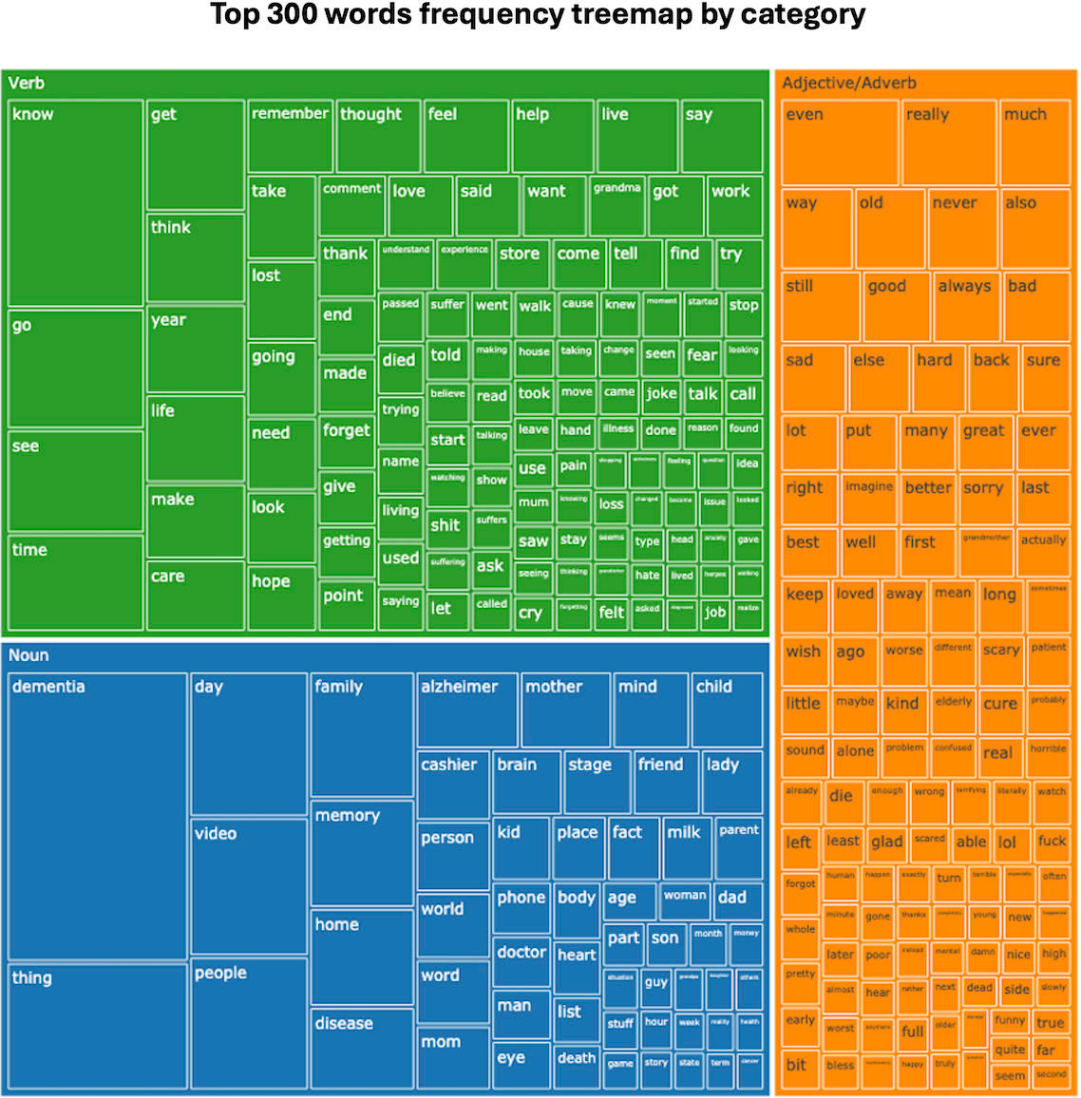
Top 300 words frequency treemap categorized by part-of-speech. The visualization groups the most frequently used words from YouTube comments into verbs (green), nouns (blue), and adjectives or adverbs (orange). The size of each word reflects its frequency.

In the adjectives or adverbs category, “even,” “really,” “much,” “old,” and “still” are prominent. These words typically modify other words to express degree, quality, or timing, indicating that commenters are emphasizing the severity, persistence, or specific qualities of the experiences or events they are discussing. In addition, words like “sorry,” “bad,” “good,” “best,” “sad,” and “hard” reflect emotional responses and value judgments, indicating that many comments convey personal sentiments or evaluations of the situations being described (Figure 2).

### Topics of the Video Comments

**Figure 3.**
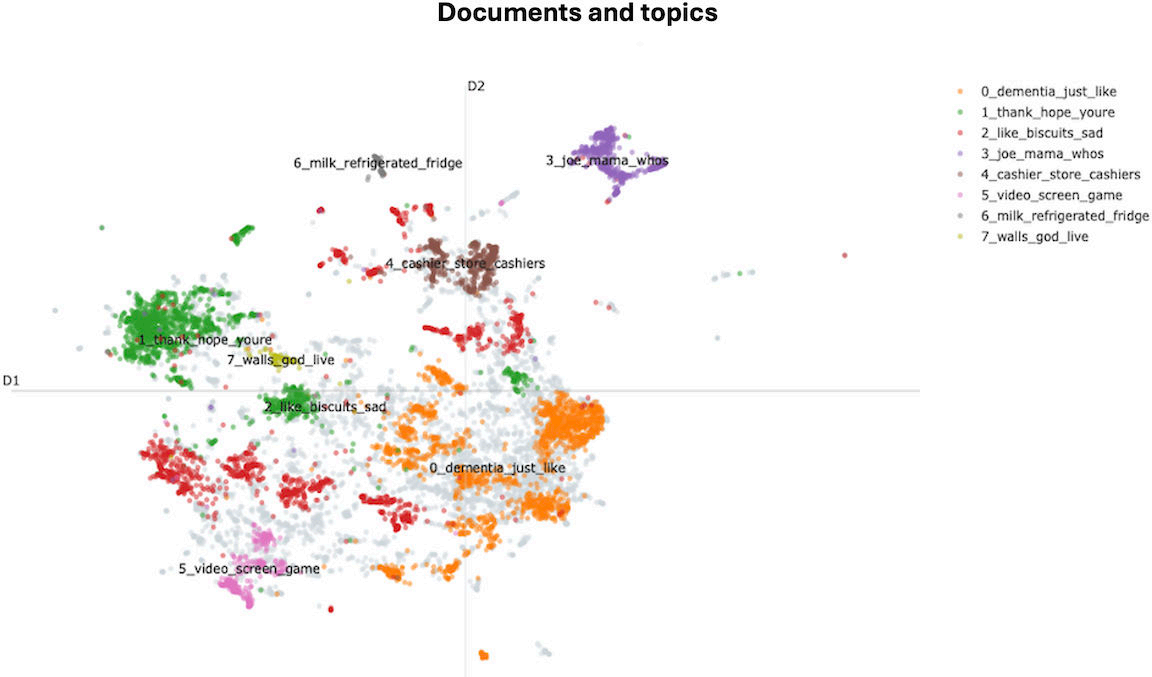
Topic distribution and clustering of “A Walk-Through Dementia” comments. Comments are distributed into 8 distinct topics, each represented by a different color. The topics are ranked from 0 to 7 based on their size, with Topic 0 (“dementia_just_like”) being the largest and Topic 7 (“walls_god_live”) the smallest.

**Figure 4.**
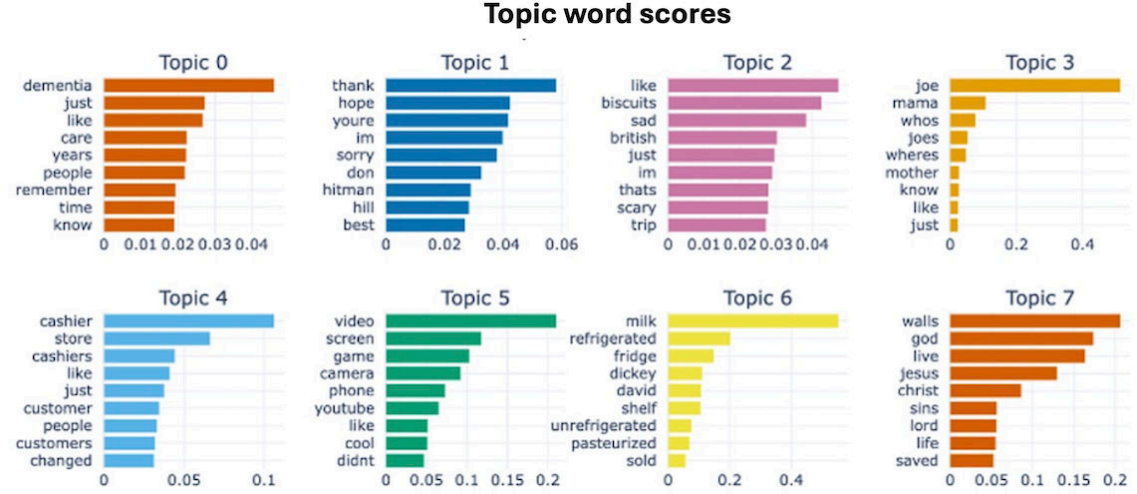
Top words by topic from “A Walk-Through Dementia” (AWTD) comments. It presents the most relevant words for each of the 8 identified topics in AWTD YouTube comments. The length of the bars after each word indicates the word’s importance within that topic. For example, the word “dementia” is the most significant word in Topic 0.

The topic modeling process identified 8 initial topics. The 2D dense clusters of these topics, visualized in Figure 3, depict the text embeddings. Each cluster, represented by a different color, corresponds to a unique topic derived from the dataset. Each point signifies a document, with spatial proximity indicating similarity between comments. The topic size decreases as the topic number increases, with Topic 0 being the largest, comprising 2040 comments. This topic is labeled “dementia_like_just” by BERTopic. The most representative words characterizing each topic are shown in Figure 4. For instance, the top 5 words in Topic 7 are “walls,” “god,” “live,” “jesus,” and “christ,” indicating that this topic revolves around religious discussions.

After manually reviewing the 8 topics and content of comments, we synthesized them into five cohesive themes reflecting viewers’ thoughts and needs: (1) the personal experience of ADRD care and expressions of empathy and support, (2) positive reactions to the VR videos, (3) challenges faced by individuals with ADRD, (4) caregivers’ role, and (5) learning from VR videos. Notably, the majority of comments reflected a deep emotional connection to the content, underscoring the significant impact of personal experiences related to ADRD. Many viewers expressed a newfound understanding and empathy toward those living with dementia and the challenges they encounter in daily life, highlighting the educational value of the immersive experience. The reviewers also discussed the roles and responsibilities of a caregiver of a person with ADRD and what the members of the general public learned from AWTD. Table 1 illustrates our topic analysis and theme development process, listing the initial topics, keywords, representative comments, and summarized themes.

**Table 1.** Overview of topic analysis and theme development. The table presents 8 topics identified by BERTopic, along with associated keywords and representative comments.

Topic ID	Keywords	Representative comment	Summarized theme
0	dementia, just, like, care, years, people, home, time, family, know	“My mom died of Alzheimer’s. It is hard when someone you love forgets where they live, who you are, what’s going on.” “That would break my heart. I guess some people get used to it and live with this sickness of one of their family members, but for an outsider, like me, reading your comment was just a tough read.”	Personal experiences of ADRD^a^ care, empathy and support
1	thank, your, I’m, hope, sorry, don, like, don’t	“I hope you are doing well, your Strength and ability to push on every day is so admirable and inspiring. Thank you for being such an amazing human being…” “I am a member of a search team, and we are called upon to search urban areas, forests, swamps and such all the time for elderly people with dementia who ‘wandered off’”	Personal experiences of ADRD care, empathy and support
2	biscuits, like, sad, British, trip, tea, I’m, scary, sugar, that’s	“Like a bad trip you have no control over” “I love the subtle details, like how there was an orange car parked by her house, but not the same type she saw earlier.” “When a person mistakes a puddle for a hole in the ground, don’t just accept that as normal behavior, look into it”	Challenges faced by individuals with ADRD
3	joe, mama, who’s, son, joes, where’s, just, like, voice, know	“who’s Joe?” “Old lady’s son” “where’s Joe?” “For me, the most terrifying part of the whole video was when Joe closed the door, leaving his mom all alone. She’s now isolated in a big home with tall stairs and couldn`t easily walk out.” “Children, you should make a cup of tea for your mum” “Dementia is often overlooked and extremely underestimated.”	Caregivers’ role
4	cashier, store, cashiers, like, just, customer, people, customers, changed, rude	“The cashier clearly has no understanding. She is rushing the poor women to pay. So rude. It’s super devastating to see this because we know they can’t stand up for themselves because of how much they struggle.” “When we put ourselves in someone’s shoes, we learn so much about others and conditions we never really paid much thought to” “I DEFINITELY need to be a more patient person with my aging mother! Thanks for posting this.” “When I was working in the nursing home, I always loved to just hold their hand, now I understand why they never refuse.”	Learning from A Walk-Through Dementia
5	video, screen, camera, game, phone, YouTube, cool, didn’t, like, realize	“This is a great 360 video that puts us right in the middle of someone’s illness” “What a powerful video. Did anyone realize that if you move your phone, it pans the camera? Very cool!” “This is so helpful, thank you! People without dementia are challenged to empathize because they don’t have the insight this video provides.” “This is so much more informative than any government add or script” “I could feel the anxiety realizing she was home alone. What a powerful message.” “I sit here reflecting on this incredible powerful and educational tool. I (My Eyes, My Heart, My Soul, My Feelings, My Spirit, My Brain) have just experience.” “I find the 360 aspect is really cool, the video is of great help to the general public in understanding the life difficulties of dementia cases.”	Positive reactions to the VR^b^ videos
6	milk, refrigerated, fridge, dickey, David, shelf, unrefrigerated, pasteurized, sold, isle	“Milk in all, 2spoon, 1s, 1/2s.; the milk wasn’t refrigerated; Why is the milk not in the fridge” “I would need to write it down for sure” “Guests can serve themselves. Or else make a list for the mother, and tape it on a cupboard door for the mother to make it easier” “They should really get her a place with no stairs, easy access to beds and rooms, cushions and pads in case she slips. Music and things to keep her distracted.”	Challenges faced by individuals with ADRD
7	god, Jesus, Christ, sins, life, saved, lord, John, repent, loves	I’m taking care of my demented grandmother… alone, 24/7. Jesus Christ it is difficult, I don’t know how long I can keep on doing this” “God bless them. I take care of dementia patients all the time at the hospital, but this sheds new light.”	Personal experiences of ADRD care, empathy, and support

a ADRD: Alzheimer disease and related dementias.

b VR: virtual reality.

## Discussion

### Principal Findings

The study evaluated AWTD, a series of free YouTube VR videos designed to raise public awareness of ADRD. At the time of data collection, AWTD was the most viewed video for the search terms “dementia” and “Alzheimer’s disease” on YouTube, boasting over 6 million views. This series also garnered the highest number of comments, totaling 12,648, compared to a similar VR video, “Coping with Alzheimer’s: Together and Apart,” which had 67,000 views and only 66 comments. The comments from viewers predominantly came from individuals with a personal connection to someone with ADRD, professionals working with affected individuals, or general audience members. This suggests that the YouTube VR videos were particularly effective in disseminating information about ADRD to a diverse and broader audience.

Our analysis identified five key themes from the comments: (1) positive reactions to the VR videos, (2) personal experience of ADRD along with expressions of empathy and support, (3) the role of caregivers, (4) challenges faced by individuals with ADRD, and (5) learning from AWTD. These findings suggest that VR has significant potential to raise public awareness of ADRD and deepen understanding of the condition, particularly through immersive and emotionally engaging experiences.

### Positive Reactions to the VR Experience and Immersive Learning

The immersive nature of VR proved highly effective in engaging viewers, allowing them to experience the challenges of ADRD firsthand, significantly enhancing their understanding of the condition. Many commenters described it as a convincing and immersive experience, both insightful and evocative, providing a powerful educational tool for ADRD care. Its appeal stemmed in part from its novelty in the field of dementia education, particularly for YouTube users, including caregivers, family members, and professionals with extensive experience in dementia care. Commenters noted that they had never encountered anything like this in their personal or professional lives. This is unsurprising, as academic literature recognizes VR as an emerging area of interest in dementia education [23,33], highlighting the need for further exploration of its applications and potential impact.

Our findings suggest that the success of the VR video lies in its ability to immerse viewers in the perspective of someone living with dementia. By wearing the VR headset, users are fully absorbed in the experience, allowing them to focus entirely on each scenario and draw connections to their personal lives or work experiences. Commenters noted how the VR video placed them directly in the midst of the illness, enabling them to feel the anxiety of a person with ADRD who is home alone. One commenter remarked, “My eyes, my heart, my soul, my feelings, my spirit, my brain have just experienced this,” emphasizing how immersion creates a profound sense of presence, making viewers feel as if they were in the shoes of someone with ADRD, inspiring empathy and support.

These findings are consistent with research from other scholars who emphasize the benefits of VR dementia education for medical students and professionals [34-36], as well as broader VR literature [37], which highlights the critical role of immersion and presence in virtual scenarios. In addition, AWTD stands out as a free VR video available on YouTube, unlike most VR content, which is typically developed and hosted on developers’ websites, requiring purchase or offering limited access [38,39]. By being widely accessible, AWTD effectively disseminates information and knowledge about ADRD to the general public, offering significant benefits for public education on the condition. This aligns with Lupton’s findings, which state that social media platforms like YouTube provide opportunities for researchers to step beyond traditional academic approaches and use innovative, free methods to share knowledge with new and diverse audiences [40].

### Role of Caregivers and Observed Gaps

The VR experience prompted viewers to reflect on the crucial role of caregivers, with many commenters noting gaps in the videos, which demonstrates how VR can stimulate critical thinking and awareness of caregiving responsibilities in ADRD. Some commenters discussed the noticeable absence of caregivers in the 3 VR videos. They expressed concern that caregivers, particularly family members, should have been more present and attentive in the depicted scenarios. Comments like “Who is Joe?” (son of an individual with ADRD), “Where is Joe?”, and “The daughter should make tea for her mother (individual with ADRD),” reflected the viewers’ belief that caregivers were missing in moments when they were needed most. In these scenarios, family members, who play a crucial role in caregiving, appeared to overlook the early signs of ADRD, such as confusion, memory lapses, and difficulty completing daily tasks. By neglecting these key symptoms, caregivers failed to provide necessary support, which may have contributed to negative outcomes for individuals with ADRD, including diminished psychosocial well-being, lowered self-esteem, and a reduced overall quality of life [41]. The VR videos can help family caregivers become more aware of their roles and responsibilities in caring for individuals with ADRD, potentially encouraging further learning and training on dementia care. This heightened awareness can lead to more proactive approaches to caregiving, allowing family members to better recognize and respond to the needs of their loved ones, ultimately improving the quality of care provided to individuals living with ADRD.

### Personal Connections and Empathy

By simulating the experiences of individuals with ADRD, VR video fostered a deeper emotional connection, encouraging viewers to express empathy and reflect on how the condition affects both patients and their families. Scenarios depicted individuals struggling to live with ADRD, allowing viewers to face challenges that they may have been previously unaware of, which were vividly portrayed through the VR experience. Commenters specifically noted moments like navigating around a puddle, which the person with ADRD believes to be a hole in the ground (street scenario), highlighting their disorientation. Other scenes—such as struggling to retrieve items from shelves and feeling rushed by a cashier (supermarket scenario) or becoming confused when asked to make tea for her daughter’s guests (AH scenario)—underscored the daily difficulties faced by individuals with ADRD. Experiencing these challenges from the perspective of someone with ADRD seemed to provide viewers with a deeper insight and better understanding of the condition’s impact.

These experiences prompted viewers to recognize how negative attitudes, impatience, or misunderstanding from others exacerbate the challenges faced by individuals with ADRD. Building a dementia-friendly or ADRD-aware community is essential to support people with ASRD to live well and carry out their daily activities. One potential approach to achieving this is to incorporate the VR video into a broader public training program, where the scenarios are reflected upon and discussed by the learners. As some commenters stated:

The cashier clearly has no understanding of ADRD. It’s super devastating to see this because we know they can’t stand up for themselves because of how much they struggle.

When we put ourselves in someone’s shoes, we learn so much about others and conditions we never really paid much thought to.

I definitely need to be a more patient person with my aging mother! Thanks for posting this.

When I was working in the nursing home, I always loved to just hold their hand, now I understand why they never refuse.

VR video is a powerful way to raise awareness of ADRD, helping the public shift their perception of the condition and learn essential skills for caring for individuals with ADRD. Similar findings regarding VR as a learning tool have been noted for medical students and professional caregivers [42,43]. The VR video therefore has a fundamental role to play in helping to create equal, inclusive communities for all, improving the actions people undertake and the language they use when interacting with those with ADRD [21].

The research findings underscore the importance of immersive learning in fostering empathy and understanding for individuals with ADRD. To build on this success, there is a clear need for more VR tools that can be used in conjunction with follow-up discussions featuring educational takeaway messages tailored for diverse audiences with varying levels of knowledge and experience regarding ADRD [44]. Creating VR programs that cater to different linguistic and cultural contexts could also address gaps in accessibility. The existing scenarios might oversimplify the condition and fail to capture the wide variability in ADRD experiences. A more diverse set of scenarios covering different stages and severities of the condition would likely provide a more nuanced understanding. The insights gained from this research can guide these discussions and aid in the development of the format and learning materials for a future VR educational program based on AWTD.

### Social Media Influence and VR in Health Communication

To contextualize the impact of VR as an educational tool, we ground our study within social media influence theory [45], which posits that social media platforms and their users shape public discourse, influence attitudes, and drive behavioral change through content creation, sharing, and interaction. Social media platforms serve as powerful conduits for disseminating health-related content, leveraging both algorithmic curation and social interactions to amplify messages. VR, as an immersive medium, engages user through both central (cognitive processing of health messages) and peripheral (emotional and experiential engagement) routes. This dual-pathway influence enhances message retention and can drive behavioral change, making VR an effective medium for public health communication.

The interactive nature of social media ensures that VR content does not exist in isolation but is actively discussed, shared, and reframed within online discourse. Algorithms prioritize highly engaging and emotionally resonant content, increasing its visibility and potential impact. User-generated discussions, as seen in the YouTube comments analyzed in this study, serve as mechanisms through which social media influence is both reinforced and coconstructed. When users share personal stories, express emotions, or react to the VR experience, they contribute to a collective conversation that reinforces common beliefs and introduces new perspectives on ADRD. This aligns with Social Media Influence theory’s assertion that online interactions shape public discourse and personal identities, ultimately contributing to the normalization or transformation of health-related attitudes and behaviors.

These theoretical perspectives allow us to interpret the thematic findings within a broader framework of digital intervention effectiveness and audience engagement. By integrating topic modeling and BERT-based analysis with media influence theory, our study not only examines the immediate impact of VR-based health communication but also contributes to a deeper understanding of how digital interventions shape public awareness and health behaviors. The methodological framework established here can be adapted and extended to different populations and varying digital health content, enabling broader applications of VR in public health communication and education.

### Limitations

One limitation of this study is the absence of demographic information about the commenters, particularly regarding their ethnicity, socioeconomic status, and professional roles. This information could provide insights into how social factors affect the experience of VR videos. In addition, as all VR video content was delivered in English, non-English-speaking populations may have been excluded, limiting the representativeness of the research sample in relation to the general public. Furthermore, the comments analyzed in this study constitute only a small portion of the total posts made in the forum, potentially failing to capture the full range of discussions surrounding ADRD. Further research could focus on gathering a more comprehensive dataset across platforms and including different languages to capture a broader perspective. Finally, this study focused exclusively on one social media platform, YouTube, and one VR video. Future research could broaden its scope by incorporating data from multiple social media platforms and a wider array of VR videos to enhance our understanding of the topic.

### Conclusion

This study used topic modeling to highlight the potential of VR videos as a powerful and immersive educational tool, offering the public a more profound understanding of the experiences of individuals living with ADRD. By fostering emotional and cognitive engagement, this approach has the potential to cultivate greater empathy and community support for those affected by ADRD. Beyond its immediate findings, this research underscores the transformative role of VR in reshaping how complex health conditions are communicated and understood. Future studies should expand on this foundation by exploring the broader applications of VR on social media platforms, such as Dementia VR Film, Beatriz Lab VR Module, and virtual reality scenarios. These tools not only have the capacity to reach wider audiences but also to drive meaningful societal change by enhancing awareness, reducing stigma, and promoting inclusive support systems for individuals with ADRD and their caregivers.

## Abbreviations

ADRD: Alzheimer disease and related dementias
AH: at home
AS: at the supermarket
AWTD: A Walk-Through Dementia
BERT: Bidirectional Encoder Representations from Transformers
LDA: latent Dirichlet allocation
NMF: nonnegative matrix factorization
VR: virtual reality
WH: walking home
